# Disease burden contributed by dietary exposure to aflatoxins in a mountainous city in Southwest China

**DOI:** 10.3389/fmicb.2023.1215428

**Published:** 2023-07-03

**Authors:** Mei Qin, Li Cheng, Yan Li, Xiaoqin Tang, Yuan Gan, Jian Zhao, Shuquan Luo, Huadong Zhang, Lishi Zhang, Jinyao Chen, Jiao Huo

**Affiliations:** ^1^West China School of Public Health and West China Fourth Hospital, Sichuan University, Chengdu, Sichuan, China; ^2^Chongqing Center for Disease Control and Prevention, Chongqing, China; ^3^Department of Preventive Health Care, Sichuan University Hospital, Chengdu, Sichuan, China; ^4^Food Safety Monitoring and Risk Assessment Key Laboratory of Sichuan Province, Chengdu, Sichuan, China

**Keywords:** aflatoxin, dietary exposure, disease burden, disability adjusted life year, hepatocellular carcinoma

## Abstract

**Introduction:**

Aflatoxins (AFT) identified as a Group 1 human carcinogen naturally contaminate various types of food and could increase the risk of hepatocellular carcinoma (HCC) through dietary intake. Chongqing municipality is located in Southwest China with subtropical monsoon climate which is conducive to AFT contamination in crops. However, the burden of HCC caused by the dietary exposure of the population in Chongqing to AFT has not been quantified.

**Methods:**

The burden of HCC was estimated in terms of Disability Adjusted Life Year (DALY) using FDA-iRISK software. Dietary exposure to AFT in three food categories including grain and its products, nuts and seeds, and spices was assessed.

**Results:**

The lifetime average daily dose (LADD) of AFT exposure for the population ranged from 2.40 to 8.25 ng/kg bw/day and 9.51 to 15.10 ng/kg bw/day at the mean and heavy (P95) AFT contamination levels, respectively. Among the three food categories, grain and its products contributed most to AFT exposure of the population. The estimated DALYs related to HCC induced by AFT were 162,000–556,000 and 641,000-1,020,000; the DALY rates were 6.47–22.20 and 25.59–40.72 per 100,000 persons per year; and the population attribution fractions (PAF) were 1.68–5.78% and 6.66–10.60%.

**Discussion:**

Although the burden of HCC caused by dietary AFT was estimated to be relatively low among the population, the overall health burden might be underestimated owing to the uncertainties of this dataset. Thus, the overall health burden associated with AFT intake should still be of concern in further studies.

## 1. Introduction

Aflatoxins (AFT) are a group of toxic secondary metabolites primarily produced by *Aspergillus flavus and Aspergillus parasiticus* (Moss, 2002). AFT contaminating can occur in a wide variety of food crops including cereals, legumes, oilseeds, nuts and spices at any stage of food production (pre-harvest, post-harvest, drying and storage) (Winter and Pereg, 2019; Gichohi-Wainaina et al., 2021). Both epidemiological surveys and animal experiments evidenced that AFT can damage human and animal liver tissues to cause hepatocellular carcinoma (HCC) and even death (Wu et al., 2014). The International Agency for Research on Cancer (IARC) evaluated the carcinogenicity of naturally occurring AFT (AFB_1_, AFB_2_, AFG_1_ and AFG_2_) for humans as Group 1 human carcinogen (IARC, 2012). In nations worldwide with high risk of HCC, there are significantly synergistic hepato-carcinogenic effects caused by AFT and hepatitis B virus (HBV) infection (Shimakawa et al., 2016; Yang et al., 2019). A meta-analysis estimated the summary odds ratio (OR) of HCC risk from both AFT exposure and HBV was 54.1, while the summary ORs on HCC risk from AFT exposure alone and chronic HBV infection alone were 5.91 and 11.2, respectively, (Liu et al., 2012). In view of the carcinogenicity and widespread contamination of AFT, and it can be believed that almost all AFT exposure results from human diet, the risk assessment and risk management of AFT intake have been gaining global visibility. Joint Food and Agricultural Organization/World Health Organization (FAO/WHO) Expert Committee on Food Additives (JECFA) has evaluated AFT for several times at the thirty-first, forty-sixth, forty-ninth, fifty-sixth, sixty-eighth and eighty-third meetings (JECFA, 1987, 1997, 1998, 2002, 2007, 2017). JECFA pointed out that dietary exposure to AFT should be reduced to the lowest feasible level in order to minimize potential HCC risk in humans. For the individuals with high prevalence of HBV surface antigen-positive (HBsAg^+^) and high intake of AFT, the reduction of AFT intake would benefit the human health.

The burden of disease is an indicator of the health and economic impact of illness, injury and early death on societies and countries (Devleesschauwer et al., 2014). The Disability Adjusted Life Year (DALY) is a standard metric to express the burden of disease measuring the healthy life years lost due to a disease or injury. It can be used to predict the health burden of foodborne disease (Devleesschauwer et al., 2015). The WHO has estimated the global burden associated with exposure to AFT to be 21,757 (8,967-56,776) HCC cases, resulting in 19,455 (7,954-51,324) deaths and 636,869 (267,142-1,617,081) DALYs (Gibb et al., 2015). Liu and Wu calculated the global AFT-related HCC cases accounted for 4.6–28.2% of the total HCC cases each year. The most heavily afflicted parts of the world were sub-Saharan Africa, Southeast Asia and China, among which China accounted for 12.1–28.9% (Liu and Wu, 2010). However, little is known regarding the disease burden of HCC caused by foodborne AFT exposure in China. Most studies on AFT risk assessment mainly focused on dietary exposure to AFT in local population without exploring the associated disease burden. Chen et al. investigated the disease burden of HCC caused by AFT intake through DALY calculation in different areas of China; however, they just assessed two kinds of food (peanuts, corn and their products) which might underestimate the burden of disease (Chen et al., 2022).

Environmental factors such as temperature, humidity, soil and storage conditions affect fungal growth and, consequently, the occurrence of AFT in foods (Jallow et al., 2021). The aflatoxin-producing molds are isolated from a wide range of climate zones, but are more frequently found between latitudes 16° and 35° in warm climate zones (i.e., tropical and subtropical zones) (Torres et al., 2014; Mao et al., 2016). Wang et al. pointed out that high-incidence areas of HCC in China generally had a warm and humid climate (Wang et al., 1983). The southwestern region of China includes Sichuan Province, Chongqing Municipality, Yunnan Province, Guizhou Province, and the Tibet Autonomous Region. Yunnan and Guizhou provinces are located in the Yungui Plateau, while the Tibet Autonomous Region is situated in the Qinghai-Tibet Plateau, both of which experience relatively lower temperatures and low humidity, and are considered low-incidence areas for HCC in China (Chen et al., 2008). In contrast, the incidence rate of HCC is relatively higher in the Chongqing-Sichuan region in Southwest China. Because of climatic diversity in China whose latitude and longitude span more than 20°, there are five different climate regions in China. The subtropical monsoon climate in Chongqing municipality (28°-32° north latitude) located in Southwest China favors the reproduction and virulence of aflatoxin-producing fungi (Wu et al., 2016; Sun et al., 2017). No study regarding the association between AFT exposure and HCC in the local population has been found in other southwestern regions of China. However, a case–control study conducted in Chongqing revealed that AFT is an independent risk factor for HCC in the population of Chongqing (Zheng et al., 2017). In addition, consumption of grain and its products, nuts and seeds, and spices is common in the dietary pattern of the population in Chongqing. Therefore, to explore the dietary AFT intake and associated health risk of the population, this study evaluated the lifetime dietary exposure to AFT and burden of HCC based on consumption data and AFT contamination data of three food categories (grain and its products, nuts and seeds, and spices) in Chongqing municipality of China.

## 2. Materials and methods

### 2.1. Contamination data

According to the sampling guideline outlined in the China Food Safety Risk Monitoring Program, a range of factors is taken into account when determining the sampling sites and sample numbers. These factors include food production, financial situation, monitoring capacity, dietary characteristics, degree of economic development, population density, geographical distribution, and other relevant considerations specific to each region. The sampling time and monitoring frequency of various monitoring samples were determined based on the characteristics of monitoring substances and their relationship with seasons, the requirements of national food safety risk assessment. A total of 694 samples of grain and its products, nuts and seeds, and spices were collected from various retail outlets (supermarkets, shops and market stalls) in different areas of Chongqing in 2012–2021. Grain and its products include rice, corn, wheat and their products; nuts and seeds include peanuts, sunflower seeds, pine nuts and their products; and spices are mainly pepper and its products.

All samples were delivered to the laboratories during 1 week and stored under ventilated and dry conditions until analysis. The levels of total aflatoxin content in samples including AFB_1_, AFB_2_, AFG_1_ and AFG_2_ were analyzed by using high-performance liquid chromatography (HPLC) or high-performance liquid chromatography tandem mass spectrometry (HPLC-MS/MS) following the China National Food Safety Standard GB/T 18979–2003, GB/T 5009.23–2006 and GB 5009.22–2016 (GAQSIQ, 2003; MOHC, 2006; NHFPC, 2016). All laboratories have passed the provincial laboratory quality certification and were requested to validate the test methodology for quality control prior to formal sample analysis. The limits of detection (LODs) among laboratories ranged from 0.006 to 0.5 μg/kg which met the requirements in the above-mentioned China National Food Safety Standards. According to the processing principle proposed by the Global Environment Monitoring System-Food Contamination Monitoring and Assessment Program (GEMS/Food), all non-detected results were replaced with 0 and LOD to produce the lower bound estimate (LB) and the upper bound estimate (UB) respectively (WHO, 1995).

### 2.2. Consumption data

The dietary consumption data was obtained from the survey data of Chongqing municipality in China Health and Nutrition Survey of 2018. A total of 979 subjects were obtained from 6 survey sites in Chongqing including Shapingba, Nanan, Dazu, Fengjie, Jiangjin and Qijiang districts and counties. The consumption data (including all foods eaten at and out home) was collected using three consecutive 24-h dietary recall by well-trained dietary staff *via* face-to-face interviews. For subjects under 7 years old and over 75 years old, dietary information came from adult family members. The food consumption within each food category was summed up for each investigated person to match with the occurrence data of each food category.

### 2.3. Risk-assessment methods

**Figure 1:**
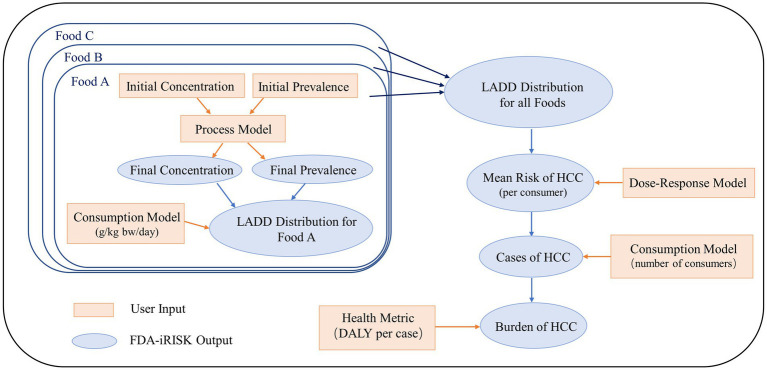
Schematic of the technical route of the chronic exposure for aflatoxin-Multifood scenario used in FDA-iRISK 4.2. LADD refers to lifetime average daily dose. HCC refers to hepatocellular carcinoma. DALY refers to Disability Adjusted Life Year.

FDA-iRISK (iRISK) is a web-based risk-assessment system developed by the U.S. Food and Drug Administration (FDA) that intended for relatively rapid assessment of the risks and public health burden associated with hazards in food (Chen et al., 2013; FDA/CFSAN, 2021). This tool mainly consists of four modules: Hazard, Food, Process, and Risk Scenario. Firstly, the Hazard module describes the relationship between AFT and HCC with a dose–response model and health metric. For the Food module, food type and consumption model were both required to estimate the average daily consumption of aflatoxin-contaminated food in any life stages. In the Process module, the processes through which the prevalence and concentration of AFT in food units change at various steps in the food chain would affect the final AFT contamination level in food. Finally, the Risk Scenario module combines three above-defined modules to simulate different risk scenarios to compute and estimate the disease burden of dietary exposure to AFT. The computed risk scenario in the present study is defined as the chronic exposure for single hazard with multiple foods scenario. The schematic of the technical route is shown in Figure 1, and the key input parameters are listed in Table 1.

**Table 1:** The main modules of FDA-iRISK 4.2 and key input parameters.

Module	Parameter	Input value
Hazard	Hazard type	Chemical
	Hazard name	Aflatoxins
	Dose response model	
	Exposure type	Chronic
	Response type	Linear by slope factor
	Slope factor	0.00017 ^a^
	Health metric (DALY per case)	12.37 ^b^
Food	Food type	Grain and its products, Nuts and seeds, Spices
	Consumption model	
	Number of consumers	32,054,159 ^c^
	Gender ratio	Male 50.55%，Female 49.45% ^c^
	Life stages	2–6, 7–17, 18–65 and 66–78.15 years old
	Average daily consumption	Linear empirical distribution (g/kg bw/day)
Process	Initial unit mass	1.0 kg (Fixed value)
	Initial prevalence ^d^	100% (Fixed value)
	Initial contamination	See Table 2 (Fixed value)
	Process model	No change
Risk Scenario	Single hazard, Multifood, Chronic exposure, Whole-group consumption	

a The inverse of the amount of AFT intake corresponding to a 10% increase in the lifetime risk of HCC was estimated using the aflatoxin carcinogenic potency derived by JECFA combined with the average life expectancy (78.15 years) published in the Report of Chongqing Residents’ Health Status in 2020.

b The DALY number of each HCC case was calculated based on the data in the China Cancer Registry Annual Report of 2019.

c The number and gender ratio of consumers obtained from the 7th National Census Bulletin of Chongqing Municipality.

d The prevalence represents the proportion of food units that are contaminated with AFT.

#### 2.3.1. Hazard module

##### 2.3.1.1. Dose–response model

The cancer slope factor (SF) of “Liner by Slope Factor” model describes the dose–response relationship between AFT exposure and HCC incidence (Wang et al., 2019; FDA/CFSAN, 2021). The SF was derived from cancer potency factor (PF) proposed by JECFA. In the case of the dietary exposure level of AFB_1_ as 1 ng/kg bw/day, JECFA estimated AFB_1_ carcinogenic potencies which corresponded to 0.3 cancer cases/year per 100,000 subjects in HBsAg-positive individuals. For HBsAg-negative individuals, the potency estimate was 0.01 cancer cases/year per 100,000 subjects (JECFA, 1998). In our present study, the carcinogenic potency of AFT (the sum of AFB_1_, AFB_2_, AFG_1_ and AFG_2_) was conservatively estimated to equal to AFB_1_. Considering the HBsAg^+^ prevalence rate in the population of Chongqing, the PF and SF were calculated as follow:


$$PF=P_{\mathrm{HBsAg}+}\times 0.3+\left(1-P_{\mathrm{HBsAg}+}\right)\times 0.01$$

$$SF=1÷\frac{100,000\;\mathrm{persons}\times 10\%}{PF\times L}$$

where P_HBsAg+_ was the HBsAg-positive rate as 4.18% among the population of Chongqing according to the Chongqing Sero-epidemiological Survey of 2015 (Kuang et al., 2018); 10% referred to a 10% increase in the lifetime risk of HCC; L was the lifespan of the targeted population.

The PF was calculated as 0.022 cases/100,000 persons/year in the context of 1 ng/kg bw/day AFT intake. It is equivalent to a 10% increase in the lifetime risk of HCC of the general population in Chongqing ingesting 5,816 ng AFT/kg bw/day, which means the SF is 0.00017 (EFSA, 2007). The lifespan of the population was calculated as 78.15 years of the average life expectancy published in the Report of Chongqing Residents’ Health Status in 2020.

##### 2.3.1.2. Health metric

The health burden of foodborne disease was measured using DALY (Havelaar et al., 2015). DALYs express the sum of years of life lost (YLLs) due to premature mortality and years of healthy life lost due to disability (YLDs) adjusted for the severity of a disease or health condition (Murray and Lopez, 1997). Chen et al. has calculated that the DALY number of each HCC case in China was 12.37 based on the data obtained from the China Cancer Registry Annual Report of 2019 (NCCR, 2020; Chen et al., 2022). And finally, the amount of aflatoxin-associated HCC cases multiplied by 12.37 equals the total DALYs.

#### 2.3.2. Food module

##### 2.3.2.1. Food type

Three food categories easily contaminated with AFT including grain and its products, nuts and seeds, and spices were assessed in our present study.

##### 2.3.2.2. Consumption model

As different foods will be consumed by different fractions of the population in each life stage, the distribution describing the consumption level will necessarily include a proportion of consumers with zero consumption (FDA/CFSAN, 2021). As such, the cumulative empirical distribution is available in iRISK to describe consumption patterns for multi-food scenarios. Since there were only 5 subjects under 2 years old in our consumption survey, and the average life expectancy in Chongqing municipality was 78.15 years, the targeted age groups (life stages) focused on the population described and computed in iRISK were 2–6 years old, 7–17 years old, 18–65 years old and 66–78.15 years old. The values of daily consumption in grams per kilogram of body weight for the life stages were required to input in the form of liner empirical distribution. iRISK modeled chronic exposure by drawing a single value randomly from the consumption distribution of each age group to simulate a large number of individual lifetime exposure patterns that were possible within the population of consumers. In this way, the distinct variants of food exposure over the course of the lifespan were condensed into a single value representing the lifetime average daily dose (LADD). The calculation and examples of LADD are presented in Supplementary Tables S1, S2.

#### 2.3.3. Process module

The process module combined the hazard module with the food module to calculate the AFT intake of the target population. It could estimate the impact of various interventions applied at specific steps in the farm-to-table continuum on AFT contamination. In this study, AFT content was uniformly distributed in the food, and both the mass of food and AFT content were assumed to be unchanged throughout the whole process (Wang et al., 2019). That is, the proportion of food contaminated with AFT per unit mass was 100% (initial and final prevalence) either before or after the process module running. Therefore, the setting value of unit mass had no effect on AFT contamination level in food and was defined as a fixed value of 1 kg in this study.

#### 2.3.4. Risk scenario module

The risk scenario module combines different scenarios from the hazard, food and process modules to estimate the burden of foodborne disease. The burden of HCC caused by dietary exposure to AFT was calculated as follows:


$$\mathrm{DAL}Y_{\mathrm{total}}=\mathrm{DAL}Y_{HCC}\times \mathrm{Case}$$

$$\mathrm{Case}=P_{\left(\mathrm{LADD}|SF\right)}\times N$$

$$P_{\left(\mathrm{LADD}|SF\right)}=\mathrm{LADD}\times SF\times 100\%$$

$$\mathrm{LADD}=\overset{n}{\underset{i=1}{\sum }}\mathrm{LAD}C_{i}\times C_{i}\times P_{i}=\overset{n}{\underset{i=1}{\sum }}\mathrm{LAD}D_{i}$$

$$\mathrm{LAD}C_{i}=\frac{\sum_{j=1}^{m}A_{j}\times Y_{j}}{\sum_{j=1}^{m}Y_{j}}$$

where *DALY_total_* referred to the total DALY number in lifetime; *DALY_HCC_* was the DALY number per HCC case; *Case* was the total amount of HCC cases; *N* was the number of consumers; *P _(LADD|SF)_* was the mean lifetime risk probability of HCC per consumer (%); *SF* was cancer slope factor; *LADD* was lifetime average daily dose for AFT intake (ng/kg bw/day); *LADC_i_* was lifetime average daily consumption amount of food category i (g/kg bw/day); *LADD_i_* was lifetime average daily dose amount of AFT intake from food category i (ng/kg bw/day); *C_i_* was the final AFT concentration of food category i (μg/kg); *n* was the number of food categories; *P_i_* was the final AFT prevalence of food category i (%); *A_j_* was the daily consumption per kilogram of body weight of life stage j (g/kg bw/day); *Y_j_* was the time span of life stage j (years); *m* was the number of life stages.

#### 2.3.5. DALY rate and population attributable fraction

The DALY rate and population attributable fraction (PAF) were also calculated for estimating the health burden and incidence of aflatoxin-related HCC. Taking into account the number of consumers, lifetime and overall annual HCC incidence, the DALY rate and PAF were calculated as follows:


$$\mathrm{DALY}\;\mathrm{rate}=\frac{\mathrm{DAL}Y_{\mathrm{total}}}{N\times L}\times 100,000$$

$$PAF=\frac{\mathrm{Case}\times 100,000}{N\times L\times R_{\mathrm{total}}}\times 100\%$$

where *DALY rate* referred to the annual DALY number per 100,000 persons (DALY/100,000 persons/year); *R_total_* was the annual all-cause HCC incidence (cases/100,000 persons/year).

### 2.4. Statistical analysis

SPSS 25.0 (IBM Corporation, Armonk, NY, United States) and R 4.2.1 (R Foundation for Statistical Computing, Vienna, Austria) were used for statistical analysis. Differences in AFT contamination among food categories were compared using the Kruskal-Wallis H-test at the 0.05 level (2-tailed). Findings were considered significant at *p* value <0.05. The dietary exposure and health burden of disease were estimated by using FDA-iRISK 4.2 (FDA/CFSAN, College Park, Maryland, United States) software.

## 3. Results

### 3.1. Contamination levels of AFT in three food categories

Table 2 shows the AFT contamination levels in grain and its products, nuts and seeds, and spices in Chongqing municipality of China. The total AFT detection rate of all the three food types was 17.4%, among which the highest AFT detection rate of 20.4% was found in nuts and seeds, followed by grain and its products (18.0%). In the case of the mean, median, P95 and maximum AFT contamination levels, grain and its products were more seriously contaminated with AFT than the other two food categories. The mean AFT concentration in grain and its products ranged from 0.61 to 2.08 μg/kg (LB-UB) which was 4–15 times higher than that in the other food categories, and the maximum concentration of AFT (37.10–38.30 μg/kg) was approximately 15-fold of that in the others.

**Table 2:** AFT contamination levels in three food categories in Chongqing municipality of China (μg/kg).

Food category	Number	Positive samples (%)	Mean		Median		P95		Max	
			LB^a^	UB^b^	LB	UB	LB	UB	LB	UB
Grain and its products	428	77 (18.0%)	0.61	2.08	0.00	1.25	2.41	3.80	37.10	38.30
Nuts and seeds^*^	152	31 (20.4%)	0.04	0.48	0.00	0.45	0.12	1.40	2.35	2.61
Spices^*^	114	13 (11.4%)	0.05	0.53	0.00	0.60	0.34	1.40	2.00	2.90
Total	694	121 (17.4%)	0.39	1.48	0.00	0.46	1.11	3.80	37.10	38.30

a Lower bound estimate: the non-detected results were assigned to 0.

b Upper bound estimate: the non-detected results were assigned to LOD.

^*^The UB values of AFT concentration were compared with grain and its products, *p* < 0.05.

### 3.2. Consumption data of three food categories

As presented in Table 3, all subjects ingested grain and its products, while a certain proportion of consumers in each age group had zero intake of spices, among which about 50% consumers aged 2–6 years consumed 0 g spices/kg bw/day. It is worth noting that the percentage of consumers ingested nuts and seeds was significantly low, with over 80% in all age groups had no consumption of nuts and seeds. The daily consumption level for grain and its products was the highest in each age group ranged from 0.43 to 24.53 g/kg bw/day, then followed by nuts and seeds (0.00–3.72 g/kg bw/day). The intake of spices was much lower at 0.00–0.49 g/kg bw/day.

**Table 3:** Consumption levels of three food categories for different age groups in Chongqing municipality of China.

Age group (years old)	Grain and its products			Nuts and seeds			Spices		
	% Consumers consumed 0 g/kg bw/day	Range of daily consumption (g/kg bw/day)	Mean daily consumption (g/kg bw/day)	% Consumers consumed 0 g/kg bw/day	Range of daily consumption (g/kg bw/day)	Mean daily consumption (g/kg bw/day)	% Consumers consumed 0 g/kg bw/day	Range of daily consumption (g/kg bw/day)	Mean daily consumption (g/kg bw/day)
2–6	0.0%	1.49–16.22	6.53	88.5%	0.00–3.72	0.14	48.2%	0.00–0.32	0.07
7–17	0.0%	0.89–24.53	5.11	91.3%	0.00–2.92	0.05	23.1%	0.00–0.49	0.08
18–65	0.0%	0.43–12.01	3.51	84.8%	0.00–2.78	0.07	13.5%	0.00–0.24	0.06
>65^a^	0.0%	0.60-11.01	3.63	85.7%	0.00–2.05	0.07	35.6%	0.00–0.22	0.05

a To match the age groups (life stages) in iRISK, the subjects aged over 78.15 years (the average life expectancy) were counted as 78.15 years old and included in the final life stage of iRISK (66–78.15 years old).

### 3.3. Dietary exposure to AFT

**Figure 2:**
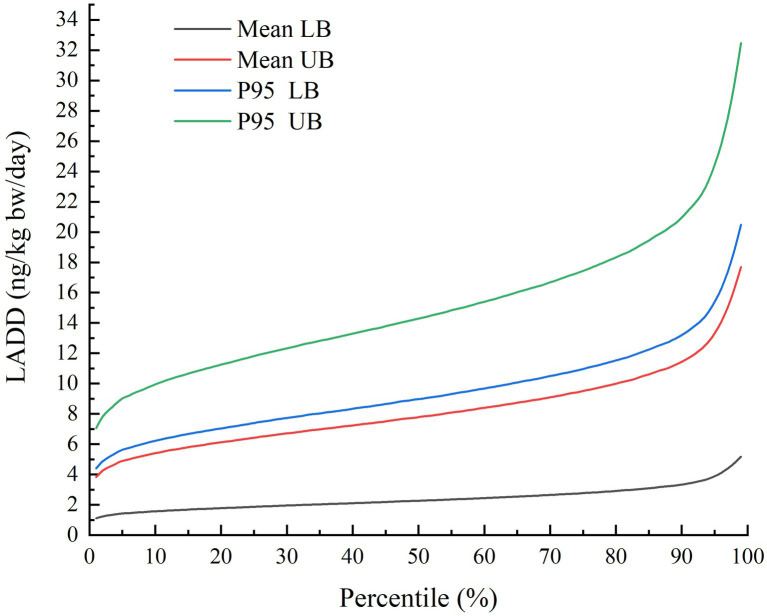
Percentile distributions of simulated LADD of AFT for the whole-group population in Chongqing municipality of China using FDA-iRISK 4.2.

As shown in Figure 2 and Table 4, the percentile distributions of LADD for the population of Chongqing were calculated by simulating using the iRISK tool based on consumption data and occurrence data of three food categories. At the mean AFT contamination level in three food categories, the LADD ranged from 1.11 to 5.18 ng/kg bw/day (LB) and 3.83 to17.69 ng/kg bw/day (UB). Meanwhile, the high percentile (P95) of AFT concentration caused much higher LADD of 4.41–20.48 ng/kg bw/day (LB) and 7.05–32.48 ng/kg bw/day (UB). Detailed exposure percentile results output from iRISK are shown in Supplementary Table S3. The representative values of LADD at the mean AFT contamination level were 2.40 ng/kg bw/day (LB) and 8.25 ng/kg bw/day (UB).

**Table 4:** AFT exposure levels and burden of HCC for the whole-group population in Chongqing municipality of China.

AFT contamination level		Representative value of LADD (ng/kg bw/day)	Lifetime risk of HCC per person (%)	HCC case	DALY_total_	DALY rate (per 100,000 persons per year)	PAF (%) ^a^
Mean	LB	2.40	0.041	13,100	162,000	6.47	1.68
	UB	8.25	0.140	44,900	556,000	22.20	5.78
P95	LB	9.51	0.162	51,800	641,000	25.59	6.66
	UB	15.10	0.257	82,500	1,020,000	40.72	10.6

a The PAF was calculated based on the overall liver cancer incidence (31.05 cases/100,000 persons/year) obtained from Chongqing Cancer Registry Annual Report of 2018.

### 3.4. Disease burden of AFT exposure

In Table 4, the representative values of LADD at the mean AFT contamination level (LB and UB) resulted in 13,100 and 44,900 HCC cases, respectively. In addition, the total corresponding DALY numbers were 162,000 and 556,000; and the DALY rates were 6.47 and 22.20 per 100,000 persons per year. Given all the risk factors of liver cancer, the foodborne AFT intake from the food categories discussed in our present study accounted for 1.68 and 5.78% (PAF) of the overall annual liver cancer incidence. The AFT intake and associated disease burden resulted from AFT contamination at the high percentile (P95) level were approximately 2–4 times higher compared with those induced by the mean AFT contamination level.

## 4. Discussion

In this study, the LADD of AFT exposure from grain and its products, nuts and seeds, and spices was estimated in the population of Chongqing municipality in China. As stated above, at the mean and P95 contamination levels of AFT in three types of food, the LADD for the population were 2.40–8.25 ng/kg bw/day (LB-UB) and 9.51–15.10 ng/kg bw/day (LB-UB), respectively, which were lower than the estimated mean AFT intake of 8.3 ng/kg bw/day and 24.9 ng/kg bw/day for the Chinese adults and children in Yangtze Delta region of China (Li et al., 2014). The reason may lie in the average daily consumption per kg bw, which was calculated based on the daily consumption *per capita* of 0.402 kg divided by 60 kg bw for adults and 20 kg bw for children, was higher than that in our study. As for other countries of Asia, a calculated daily intake of AFB_1_ for the Korean population fell into the range of 0.0640–0.3612 ng/kg bw/day (Lee et al., 2009). The dietary exposure to AFB_1_ from all foods contaminated was only 0.003–0.004 ng/kg bw/day even though at the 95th percentile level of consumer population in Japan (Sugita-Konishi et al., 2010). Nevertheless, Malaysia estimated the dietary exposure to AFB_1_ to range from 24.3 to 34.0 ng/kg bw/day in the adult Malaysian diet (Chin et al., 2012). Additionally, the exposure to AFT shown in our study was higher compared with that in EFSA’s report of 2020. In that report, the dietary exposure to AFB_1_ from multiple foods in European population was estimated to be 0.08–6.95 ng/kg bw/day and 0.35–14.01 ng/kg bw/day for all age groups at mean and P95 exposure levels, respectively (EFSA Panel on Contaminants in the Food Chain (CONTAM) et al., 2020). Such differences in AFT intake levels could be concerned with the temperate climate of most of the EU regions and the subtropical climate of Chongqing. In contrast, dietary AFT exposure estimates in Africa (1.4–850 ng/kg bw/day) (Andrade and Caldas, 2015) were significantly higher than our findings. A survey conducted in sub-Saharan Africa revealed that the human dietary exposure to AFT, ranging between 4 and 526 ng/kg bw/day, also highlighted this discrepancy (Ingenbleek et al., 2020). The aforesaid findings agreed with the findings that the higher HCC risk occurred in the populations of sub-Saharan Africa, Southeast Asia and China, which were located in tropical and subtropical regions exposed to climate conditions favorable for AFT contamination (McGlynn et al., 2015; Sayiner et al., 2019).

Although it was not appropriate to establish a tolerable daily intake in view of the genotoxic properties of AFT, JECFA indicated that the risk of one extra cancer case per million people per year was acceptable (JECFA, 1998). The risk of extra HCC cases obtained for the target population evaluated in this study exceeded the above recognized unacceptable cancer risk. Our assessment revealed that the attributable risk of HCC caused by AFT intake in Chongqing municipality of China was estimated to be 1.68–5.78% at the mean contamination level of AFT in food. It is slightly below the global attributable risk of AFT-associated HCC (4.6–28.2%) reported by Liu and Wu (2010). Also, this is the first study to estimate the burden of disease associated with the current AFT exposure for the population in Chongqing in terms of DALY. The mean DALY rate of dietary AFT exposure for the population ranged from 6.47 to 22.20 per 100,000 persons per year (LB-UB), and increased to 25.59–40.72 per 100,000 persons per year at the 95th percentile of AFT contamination level. In 2022, Chen et al. evaluated the disease burden induced by dietary exposure to AFT in different areas of China. The national-level estimates of average DALY rate (1.53 per 100,000 persons per year) and PAF (0.69%) were both lower comparing with our results. In particular, the DALY rate of Chongqing municipality (0.01 per 100,000 persons per year) was far below our estimate of a mean of 6.47–22.20 per 100,000 persons per year. The discrepancy may be owing to the fact that only peanuts and peanut oil among oil in Chongqing municipality were included in that study, and the attributable exposure was merely 0.025 ng/kg bw/day (Chen et al., 2022). An evaluation conducted in Taiwan of China indicated the total annual DALYs caused by AFT contamination was 4,110, equivalent to 24.63 DALYs per 100,000 persons per year, which was higher than our results (Wang et al., 2019). The remarkable thing was that the HBsAg-positive rate of Taiwanese (17.3%) far above that of the population in Chongqing (4.18%). A WHO report by the Foodborne Disease Burden Epidemiology Reference Group (FERG) estimated the burden of disease related to AFT intake for each of 14 subregions (Havelaar et al., 2015; WHO, 2015). Among them, African Region D (AFR D) revealed the highest median DALY rate of 28 per 100,000 persons per year, followed by Western Pacific Region B (WPB R, including China) with a median DALY rate of 17 per 100,000 persons per year, which was close to our estimates. The two subregions with the lowest median DALY rate were Region A of the Americas (AMR A) and European Region A (EUR A), with only 0.04 and 0.3 DALYs per 100,000 persons per year, respectively. Comparisons with national studies showed the similar findings. Ricardo Assuncao et al. estimated the burden of disease associated to the current AFT exposure for Portuguese population to be 0.08–0.30 DALYs per 100,000 persons per year (Assuncao et al., 2018). Nevertheless, there was a strikingly high risk of AFT contamination in food ingested by Nigerian consumers, resulting in a national burden of 126.85–38,682.29 DALYs per 100,000 persons per year (Adetuniji et al., 2014). Furthermore, given the all-cause HCC incidence of the population of Chongqing reported to be 31.05 cases/100,000 persons/year (Rong et al., 2021), the overall HCC-burden was calculated to be 394.3 DALYs per 100,000 persons per year. In general, our findings suggest the current occurrence of AFT appears to be not a serious problem in Chongqing municipality of China, but it still causes certain risks concerning public health.

As is well-known, the environmental conditions of warm and high humidity are conducive to AFT-related fungal infection and toxin production (Do et al., 2020; Kerry et al., 2021). In this study, the AFT contamination in food of Chongqing was more severe than China’s national level, especially than that in northern China (Zhao et al., 2020; Yan et al., 2022). The subtropical monsoon climate of Chongqing municipality in Southwest China was reported to be more conducive to fungal reproduction and virulence versus the temperate continental climate and temperate monsoon climate in the northern China (Qin et al., 2021). Through the surveillance in our study, grain and its products in Chongqing were founded to be contaminated more seriously with AFT than nuts, seeds and spices. In addition, grain and its products account for a much larger proportion in the dietary consumption of the population in Chongqing than the other two types of food, so grain and its products can be considered to be the main food contributing to AFT exposure in the population’s diets. Chongqing municipality and Chengdu of Sichuan province, also located in Southwest China, once carried out AFT contamination surveys on unpackaged spices, and the AFT contamination levels were both higher than the values among this dataset, with median AFT content of 3.014–12.849 μg/kg and 1.1–57.1 μg/kg, respectively (Fu et al., 2015; Huang et al., 2020). This difference suggested that prolonged exposure of unpackaged food to external environment could increase the chance of AFT contamination as the lack of well-controlled storage conditions (Set and Erkmen, 2014; Naz et al., 2016). Various methods have been applied to degrade AFT, such as good agricultural practice (GAP), biocontrol, improving crop storage packaging, ozonolysis, alkali refining, ultraviolet (UV) irradiation and so on (Sudini et al., 2014; Mao et al., 2016; Parimi et al., 2018; Shabeer et al., 2022). However, it is expected that in the future the HCC-burden contributed by AFT exposure will increase due to climate change (Cotty and Jaime-Garcia, 2007; Assuncao et al., 2018). Reducing dietary exposures to the carcinogen AFT is likely to significantly reduce HCC risk, even in those already infected with HBV (Chen et al., 2013). Hence, consecutive monitoring and controlling AFT contamination in food to avoid future exposure of vast human populations to unacceptable AFT levels are essential ways to promote the food safety and human health.

Our study evaluated the lifetime exposure patterns of the population in Chongqing to AFT and firstly quantified the health burden of AFT-induced HCC. However, several uncertainty factors and limitations in the present study need to be addressed. Firstly, cancer potency for AFB_1_ was applied to ‘aflatoxin total’ because the available data could not make it possible to identify potency factors of all types of AFT. The uncertainty arising from this conservative approach should be noted, while EFSA reported that the influence of the assumption on the conclusion regarding the risk resulted from the presence of AFT in food was small (EFSA Panel on Contaminants in the Food Chain (CONTAM) et al., 2020). In the dose–response model, the derivation of the cancer potency factor (PF) only adjusted for the prevalence of hepatitis B infection in the local population and did not consider other confounding factors such as alcohol, hepatitis C infection, and consumption of raw meat. It should be noted that the population of Chongqing does not have a dietary habit of consuming raw meat, and the prevalence of hepatitis C infection is significantly lower compared to hepatitis B (Wang et al., 2007). Therefore, these two confounding factors was considered to pose little effect on the PF. However, alcohol intake is a well-recognized independent risk factor for HCC, and the lack of adjustment for it in this study might introduce some uncertainty. This would be one of the key points to address in future research. In the process module, fixed values were used for the calculations, so uncertainties ranges were not quantified due to the lack of raw data. Also, we assumed that the AFT content in food did not change throughout the whole process, but it is unclear what combined effect food processing and cooking might have had on AFT levels in consumed foods. Full studies with the farm-to-table continuum on AFT contamination are recommended in the future. Additionally, we collected samples in markets rather than in homes of residents and farmers, where storage conditions may affect aflatoxins contaminations. Total dietary studies may be conducted thereafter to reduce this uncertainty. Finally, the absent food categories in this study such as vegetable oil and beans have been the main sources of dietary exposure to AFT (Pickova et al., 2021). A full-scale estimation for the overall dietary AFT exposure of population needs to be conducted. In summary, we consider the risk assessment in our study is likely to be conservative.

## 5. Conclusion

This study presents the first disease burden estimates of HCC induced by the dietary exposure to AFT in three food categories including grain and its products, nuts and seeds, and spices, among population in a mountainous city (Chongqing municipality), in Southwest China. Comparing with nuts and seeds, and spices, grain and its products contributed most to dietary AFT exposure of the population. In terms of lifetime chronic exposure levels, the burden of HCC induced by AFT exposure among the population was relatively low in the context of all-cause liver cancer incidence. However, the health risk caused by AFT contamination should still be of concern. Given the future climate change, continuous monitoring and controlling of AFT contamination to restrict dietary exposure are recommended.

## Data availability statement

The raw data supporting the conclusions of this article will be made available by the authors, without undue reservation.

## Author contributions

MQ, JC, YL, and LZ conceived the concept. MQ, LC, XT, and YG carried out the project and collected the data. MQ analysed the data, wrote the initial draft, and revised the manuscript. JC, JH, YL, and LZ critically revised the manuscript and finalized the manuscript. JC and JH significantly contributed to review the manuscript in reply to reviewers. JZ, SL, and HZ supervised and administrated the project. All authors contributed to the article and approved the submitted version.

## Funding

This research was funded by the project of Science and Technology Department of Sichuan Province in China (no 2019YJ0020), the China Postdoctoral Science Foundation (no 2022 M710549), the Natural Science Foundation of Chongqing (no cstc2021jcyj-msxmX0479), and the First batch of Key Disciplines on Public Health in Chongqing and the Scientific and Technological Innovation Center in Chongqing.

## Conflict of interest

The authors declare that the research was conducted in the absence of any commercial or financial relationships that could be construed as a potential conflict of interest.

## Publisher’s note

All claims expressed in this article are solely those of the authors and do not necessarily represent those of their affiliated organizations, or those of the publisher, the editors and the reviewers. Any product that may be evaluated in this article, or claim that may be made by its manufacturer, is not guaranteed or endorsed by the publisher.
