# Supplementary material for: Disease burden contributed by dietary exposure to aflatoxins in a mountainous city in Southwest China
Source: Front Microbiol. 2023 Jul 3;14:1215428. doi: 10.3389/fmicb.2023.1215428 (PMC10351015; doi:10.3389/fmicb.2023.1215428)
Supplement: Supplementary file 1 [file Table_1.DOCX]

Supplementary Material

# The calculation and examples of lifetime average daily dose in FDA-iRISK 4.2

FDA-iRISK modeled chronic aflatoxins (AFT) exposure by simulating a large number of individual lifetime exposure patterns that might within the population of consumers. Each iteration of the model simulated a pattern, and the patterns varied among tens of thousands of iterations. For example, the first lifetime exposure pattern might be characterized by very high childhood and youth exposure, followed by very low subsequent exposure, while the second pattern might feature high exposure in adulthood and old age but low exposure in childhood and youth.

Consumption data in this study was input in terms of cumulative empirical distribution of daily consumption in grams per kilogram of body weight. FDA-iRISK sampled randomly a single value from the distribution for each life stage in each iteration to simulate a lifetime consumption pattern. The overall dose assigned to all life stages exposure was the lifetime average daily dose (LADD), the daily dose of the AFT ingested by the consumer (attributable to the food in question) averaged over the lifetime. The contribution of each life stage to LADD was proportional to the length of the life stage. In this way, the changing lifetime exposure was condensed into a representative value of the LADD. Then, the representative LADD was provided to the dose response model to obtain a mean risk of illness per consumer.

Our study simulated the possible lifetime exposure patterns of AFT at the mean and 95th percentile contamination levels in three types of food. Table S1 and Table S2 illustrate the LADD calculation examples resulting from iterations in which randomly sampled consumption values respectively represent the consumption patterns corresponding to high consumption level at a young age or at an older age. For the purposes of this example, the final concentration of AFT in grain and its products is 2.08 μg/kg and the final prevalence is 100%. The below iterations only represent two exposure patterns of AFT in grain and its products. The total LADD of AFT in all food categories can be obtained after simulating the LADD values of the other food categories in the same way. The formulas are as follow:

$$\boldsymbol{LADD=}\sum_{\boldsymbol{i=1}}^{\boldsymbol{n}} \boldsymbol{LADC}_{\boldsymbol{i}}\boldsymbol{\times}\boldsymbol{C}_{\boldsymbol{i}}\boldsymbol{\times}\boldsymbol{P}_{\boldsymbol{i}}\boldsymbol{=}\sum_{\boldsymbol{i=1}}^{\boldsymbol{n}} \boldsymbol{LADD}_{\boldsymbol{i}}$$

$$\boldsymbol{LADC}_{\boldsymbol{i}}\boldsymbol{=}\frac{\sum_{\boldsymbol{j=1}}^{\boldsymbol{m}} \boldsymbol{A}_{\boldsymbol{j}}\boldsymbol{\times}\boldsymbol{Y}_{\boldsymbol{j}}}{\sum_{\boldsymbol{j=1}}^{\boldsymbol{m}} \boldsymbol{Y}_{\boldsymbol{j}}}$$

where *LADD* was lifetime average daily dose for AFT intake (ng/kg bw/day); *LADC_i_* was lifetime average daily consumption amount of food category i (g/kg bw/day); *LADD_i_* was lifetime average daily dose amount of AFT intake from food category i (ng/kg bw/day); *C_i_* was the final AFT concentration of food category i (μg/kg); *n* was the number of food categories; *P_i_* was the final AFT prevalence of food category i (%); *A_j_* was the daily consumption per kilogram of body weight of life stage j (g/kg bw/day); *Y_j_* was the time span of life stage j (years); *m* was the number of life stages.

**Table S1.** Calculation of the LADD for AFT from grain and its products-Iteration 1

| Life Stage (years old) | 2 to 6 | 7 to 17 | 18 to 65 | 66 to 78.15 |
| --- | --- | --- | --- | --- |
| Consumption (*A_j_*, g/kg bw/day) | 10.52 | 6.19 | 8.07 | 5.61 |
| Time Span (*Y_j_*, years) | 5 | 11 | 48 | 12.15 |
| *LADC_i_* (g/kg bw/day) | 7.567 | | | |
| AFT concentration (*C_i_*, μg/kg) | 2.08 | | | |
| AFT prevalence (*P_i_*, %) | 100 | | | |
| *LADD_i_* (ng/kg bw/day) | 15.74 | | | |

**Table S2.** Calculation of the LADD for AFT from grain and its products-Iteration 2

| Life Stage (years old) | 2 to 6 | 7 to 17 | 18 to 65 | 66 to 78.15 |
| --- | --- | --- | --- | --- |
| Consumption (*A_j_*, g/kg bw/day) | 3.12 | 5.58 | 8.35 | 10.21 |
| Time Span (*Y_j_*, years) | 5 | 11 | 48 | 12.15 |
| *LADC_i_* (g/kg bw/day) | 7.903 | | | |
| AFT concentration (*C_i_*, μg/kg) | 2.08 | | | |
| AFT prevalence (*P_i_*, %) | 100 | | | |
| *LADD_i_* (ng/kg bw/day) | 16.44 | | | |

# Supplementary Table S3

**Table S3.** The percentile values of LADD for AFT in three food categories resulting from different lifetime exposure patterns simulated using FDA-iRISK 4.2. (ng/kg bw/day)

| **Percentile (%)** | **Aflatoxin contamination level** | | | |
| --- | --- | --- | --- | --- |
|  | **Mean LB** | **Mean UB** | **P95 LB** | **P95 UB** |
| **1** | 1.11 | 3.83 | 4.41 | 7.05 |
| **2** | 1.23 | 4.25 | 4.87 | 7.83 |
| **3** | 1.31 | 4.50 | 5.17 | 8.28 |
| **4** | 1.37 | 4.70 | 5.40 | 8.66 |
| **5** | 1.42 | 4.90 | 5.62 | 9.01 |
| **6** | 1.46 | 5.00 | 5.76 | 9.20 |
| **7** | 1.49 | 5.12 | 5.88 | 9.41 |
| **8** | 1.52 | 5.21 | 6.00 | 9.57 |
| **9** | 1.55 | 5.32 | 6.12 | 9.77 |
| **10** | 1.57 | 5.41 | 6.22 | 9.94 |
| **11** | 1.60 | 5.50 | 6.32 | 10.10 |
| **12** | 1.62 | 5.58 | 6.42 | 10.26 |
| **13** | 1.64 | 5.65 | 6.50 | 10.40 |
| **14** | 1.67 | 5.73 | 6.59 | 10.52 |
| **15** | 1.69 | 5.80 | 6.67 | 10.66 |
| **16** | 1.71 | 5.87 | 6.76 | 10.77 |
| **17** | 1.73 | 5.93 | 6.83 | 10.90 |
| **18** | 1.75 | 6.00 | 6.90 | 11.02 |
| **19** | 1.76 | 6.06 | 6.97 | 11.13 |
| **20** | 1.78 | 6.12 | 7.04 | 11.25 |
| **21** | 1.80 | 6.18 | 7.12 | 11.35 |
| **22** | 1.82 | 6.24 | 7.18 | 11.47 |
| **23** | 1.83 | 6.31 | 7.25 | 11.58 |
| **24** | 1.85 | 6.36 | 7.32 | 11.70 |
| **25** | 1.87 | 6.43 | 7.40 | 11.82 |
| **26** | 1.89 | 6.49 | 7.48 | 11.93 |
| **27** | 1.91 | 6.55 | 7.54 | 12.03 |
| **28** | 1.92 | 6.61 | 7.60 | 12.13 |
| **29** | 1.94 | 6.66 | 7.67 | 12.24 |
| **30** | 1.96 | 6.72 | 7.74 | 12.33 |
| **31** | 1.97 | 6.77 | 7.80 | 12.43 |
| **32** | 1.99 | 6.83 | 7.86 | 12.54 |
| **33** | 2.00 | 6.88 | 7.92 | 12.63 |
| **34** | 2.02 | 6.93 | 7.98 | 12.72 |
| **35** | 2.03 | 6.98 | 8.03 | 12.82 |
| **36** | 2.05 | 7.03 | 8.10 | 12.91 |
| **37** | 2.06 | 7.08 | 8.15 | 13.00 |
| **38** | 2.08 | 7.13 | 8.21 | 13.10 |
| **39** | 2.09 | 7.19 | 8.27 | 13.20 |
| **40** | 2.11 | 7.24 | 8.34 | 13.30 |
| **41** | 2.12 | 7.29 | 8.40 | 13.40 |
| **42** | 2.14 | 7.35 | 8.46 | 13.49 |
| **43** | 2.15 | 7.40 | 8.52 | 13.59 |
| **44** | 2.17 | 7.45 | 8.58 | 13.68 |
| **45** | 2.19 | 7.51 | 8.65 | 13.79 |
| **46** | 2.21 | 7.57 | 8.72 | 13.89 |
| **47** | 2.22 | 7.63 | 8.79 | 14.00 |
| **48** | 2.24 | 7.68 | 8.85 | 14.10 |
| **49** | 2.26 | 7.73 | 8.92 | 14.18 |
| **50** | 2.27 | 7.78 | 8.98 | 14.28 |
| **51** | 2.28 | 7.84 | 9.03 | 14.38 |
| **52** | 2.30 | 7.90 | 9.10 | 14.50 |
| **53** | 2.32 | 7.96 | 9.16 | 14.59 |
| **54** | 2.33 | 8.02 | 9.23 | 14.71 |
| **55** | 2.35 | 8.08 | 9.31 | 14.84 |
| **56** | 2.37 | 8.14 | 9.39 | 14.95 |
| **57** | 2.39 | 8.20 | 9.45 | 15.05 |
| **58** | 2.41 | 8.26 | 9.53 | 15.17 |
| **59** | 2.43 | 8.33 | 9.61 | 15.29 |
| **60** | 2.45 | 8.39 | 9.68 | 15.40 |
| **61** | 2.47 | 8.46 | 9.75 | 15.51 |
| **62** | 2.49 | 8.52 | 9.83 | 15.63 |
| **63** | 2.51 | 8.59 | 9.91 | 15.77 |
| **64** | 2.53 | 8.66 | 9.99 | 15.89 |
| **65** | 2.55 | 8.74 | 10.07 | 16.02 |
| **66** | 2.57 | 8.80 | 10.16 | 16.15 |
| **67** | 2.59 | 8.88 | 10.23 | 16.28 |
| **68** | 2.61 | 8.94 | 10.31 | 16.40 |
| **69** | 2.63 | 9.01 | 10.40 | 16.53 |
| **70** | 2.65 | 9.10 | 10.49 | 16.69 |
| **71** | 2.68 | 9.18 | 10.59 | 16.83 |
| **72** | 2.70 | 9.25 | 10.68 | 16.97 |
| **73** | 2.72 | 9.35 | 10.77 | 17.13 |
| **74** | 2.75 | 9.42 | 10.86 | 17.28 |
| **75** | 2.77 | 9.51 | 10.96 | 17.44 |
| **76** | 2.80 | 9.59 | 11.07 | 17.60 |
| **77** | 2.83 | 9.69 | 11.17 | 17.77 |
| **78** | 2.86 | 9.79 | 11.29 | 17.95 |
| **79** | 2.88 | 9.89 | 11.41 | 18.14 |
| **80** | 2.92 | 9.99 | 11.53 | 18.32 |
| **81** | 2.95 | 10.10 | 11.65 | 18.52 |
| **82** | 2.98 | 10.20 | 11.77 | 18.71 |
| **83** | 3.02 | 10.33 | 11.93 | 18.95 |
| **84** | 3.06 | 10.47 | 12.10 | 19.20 |
| **85** | 3.10 | 10.61 | 12.24 | 19.45 |
| **86** | 3.14 | 10.77 | 12.42 | 19.74 |
| **87** | 3.19 | 10.91 | 12.59 | 19.97 |
| **88** | 3.23 | 11.05 | 12.75 | 20.26 |
| **89** | 3.28 | 11.23 | 12.96 | 20.57 |
| **90** | 3.34 | 11.43 | 13.19 | 20.95 |
| **91** | 3.41 | 11.68 | 13.47 | 21.41 |
| **92** | 3.50 | 11.96 | 13.82 | 21.89 |
| **93** | 3.58 | 12.28 | 14.17 | 22.47 |
| **94** | 3.71 | 12.71 | 14.67 | 23.31 |
| **95** | 3.89 | 13.31 | 15.39 | 24.43 |
| **96** | 4.11 | 14.07 | 16.26 | 25.77 |
| **97** | 4.40 | 15.04 | 17.39 | 27.53 |
| **98** | 4.76 | 16.27 | 18.81 | 29.78 |
| **99** | 5.18 | 17.69 | 20.48 | 32.48 |
